# Pathogenomes of Shiga Toxin Positive and Negative *Escherichia coli* O157:H7 Strains TT12A and TT12B: Comprehensive Phylogenomic Analysis Using Closed Genomes

**DOI:** 10.3390/microorganisms12040699

**Published:** 2024-03-29

**Authors:** Anwar A. Kalalah, Sara S. K. Koenig, Peter Feng, Joseph M. Bosilevac, James L. Bono, Mark Eppinger

**Affiliations:** 1 Department of Molecular Microbiology and Immunology, University of Texas at San Antonio, San Antonio, TX 78249, USA; 2 South Texas Center for Emerging Infectious Diseases (STCEID), San Antonio, TX 78249, USA; 3 U.S. Food and Drug Administration (FDA), College Park, MD 20740, USA; 4 U.S. Department of Agriculture (USDA), Agricultural Research Service (ARS), U.S. Meat Animal Research Center, Clay Center, NE 68933, USA

**Keywords:** Shiga toxin (Stx)-producing *Escherichia coli* (STEC), O157:H7, Stx-converting bacteriophages, whole genome sequencing and typing (WGST), comparative phylogenomics, bacterial isogens, lost Shiga toxin (LST)

## Abstract

Shiga toxin-producing *Escherichia coli* are zoonotic pathogens that cause food-borne human disease. Among these, the O157:H7 serotype has evolved from an enteropathogenic O55:H7 ancestor through the displacement of the somatic gene cluster and recurrent toxigenic conversion by Shiga toxin-converting bacteriophages. However, atypical strains that lack the Shiga toxin, the characteristic virulence hallmark, are circulating in this lineage. For this study, we analyzed the pathogenome and virulence inventories of the *stx*+ strain, TT12A, isolated from a patient with hemorrhagic colitis, and its respective co-isolated *stx*− strain, TT12B. Sequencing the genomes to closure proved critical to the cataloguing of subtle strain differentiating sequence and structural polymorphisms at a high-level of phylogenetic accuracy and resolution. Phylogenomic profiling revealed SNP and MLST profiles similar to the near clonal outbreak isolates. Their prophage inventories, however, were notably different. The attenuated atypical non-shigatoxigenic status of TT12B is explained by the absence of both the ΦStx_1a_- and ΦStx_2a_-prophages carried by TT12A, and we also recorded further alterations in the non-Stx prophage complement. Phenotypic characterization indicated that culture growth was directly impacted by the strains’ distinct lytic phage complement. Altogether, our phylogenomic and phenotypic analyses show that these intimately related isogenic strains are on divergent Stx(+/stx−) evolutionary paths.

## 1. Introduction

Shiga toxin-producing *Escherichia coli* (STEC) are causative agents of severe foodborne human disease [1]. Among these, the O157:H7 lineage has emerged as one of the globally predominant pathogenic serotypes [2,3,4,5,6,7,8]. Infections with STEC O157:H7 can cause hemorrhagic colitis (HC) [9,10,11], which may lead to life-threatening complications such as the hemolytic uremic syndrome (HUS), ultimately resulting in renal failure [12,13,14,15,16].

Notable virulence determinants in STEC are the Shiga toxins [17], the lineage-specific pO157 virulence plasmid [18], and the locus of enterocyte effacement (LEE) [19,20,21], responsible for the characteristic attaching and effacing lesions [22,23]. The LEE pathogenicity island contains type III secretion system (T3SS) along with various LEE and non-LEE effectors [22,24,25]. The defining virulence hallmark is the production of the phage-borne Shiga toxin, a potent protein synthesis inhibitor [26,27], which is sufficient to cause disease [28]. This toxin is cytopathic for eukaryotic cells and specifically toxigenic to renal cells [29,30,31,32,33,34,35]. Single or multiple ΦStx-prophages can be carried, with the toxin expressed and produced after phage mobilization during the phage lytic cycle [31,36,37,38,39,40]. Various suballeles of the major *stx* alleles 1 and 2 have been described [41,42,43,44]. Among these, Stx_2a_ is highly cytopathic, with up to 400x increased toxicity in mice as compared to Stx_1a_ [11,28,45,46,47,48,49].

Serotype O157:H7 evolved from an *stx*-negative enteropathogenic *E. coli* (EPEC) O55:H7 progenitor through the toxigenic conversion of strains by Stx-phages [50,51,52,53,54,55,56,57,58,59,60,61]. However, atypical non-shigatoxigenic strains have been described in diverse STEC serotypes [52,54,62,63,64,65,66,67,68,69,70,71,72,73,74,75,76,77]. Complex genomic alterations can result in the disruption, confinement, or complete loss of the *stx* locus or entire ΦStx-prophages [68]. Such alterations may occur during routine culturing or intentionally in the laboratory through the addition of phage-mobilizing agents to the growth media [69,70,74,75,77,78].

This study analyzes the clinical O157:H7 isolate, TT12, which originated from a patient presenting with hemorrhagic colitis. When grown on selective media, the original study found that the isolates exhibited two distinct colony morphologies, designated as TT12A and TT12B [62]. Subsequent *stx* PCR-interrogation indicated that TT12A and TT12B were Stx(+) and Stx(−), respectively. Further molecular analyses suggested that these strains were isogenic, but the results were not definitive, and not informed by genome sequences. For this study, we investigated the isolates’ presumed isogenic status from a whole genome perspective making use of high-resolution comparative genomics techniques. The generation of high-quality closed genomes provided the basis for in-depth phylogenomic comparisons and allowed us to catalogue subtle strain-differentiating sequence and structural polymorphisms, which explain the atypical, non-shigatoxigenic status of strain TT12B.

## 2. Materials and Methods

### 2.1. Bacterial Strains Analyzed in This Study

Strain-associated metadata for TT12A and TT12B, along with other O157:H7 strains investigated in this study, can be found in Supplemental Table S1. The genome of TT12A was sequenced to closure in this study, while the co-isolated TT12B genome was previously sequenced by our group [68].

### 2.2. Genome Sequencing, Assembly, and Annotation

TT12A strain was cultured overnight at 37 °C with shaking at 220 rpm in lysogeny broth (LB) (Thermo Fisher Scientific, Asheville, NC, USA). The culture was then diluted to an OD_600_ of 0.03 in fresh LB medium and grown at 37 °C with shaking at 220 rpm to mid-log phase (OD_600_~0.5). Total genomic DNA (gDNA) was extracted using the QIAamp DNA Mini Kit (Qiagen, Inc., Valencia, CA, USA) according to the manufacturer’s instructions. Genomic DNA preparation was subjected to both long-read (Pacific Biosciences, Menlo Park, CA, USA) and short-read (Illumina, San Diego, CA, USA) sequencing. For long-read sequencing on the PacBio RS II platform, gDNA was sheared into 20 kb fragments using g-TUBE (Covaris, Inc., Woburn, MA, USA). The library was prepared based on the 20 kb PacBio sample preparation protocol and sequenced using P6/C4 chemistry on four single-molecule real-time (SMRT) cells with a 240 min collection time. The continuous long-read data were de novo assembled using the PacBio hierarchical genome assembly process (HGAP v.3.0) with the default parameters in SMRT Analysis (v.2.3.0), including consensus polishing with Quiver [79]. Long-reads were complemented with Illumina short-reads generated on the MiSeq platform. Paired-end libraries were prepared with the NxSeq AmpFREE Low DNA Library Kit (Lucigen, Middleton, WI, USA) with a 250 bp read length and sequenced using the MiSeq Reagent kit (v2) (500-cycle). Sequencing reads in the fastq format were imported into Galaxy [80], and the default software parameters were used for all analysis unless specified otherwise. FastQC (v.0.74 + Galaxy0) (http://www.bioinformatics.babraham.ac.uk/projects/fastqc, accessed on 10 December 2023) and Trim Galore (https://www.bioinformatics.babraham.ac.uk/projects/trim_galore/, accessed on 10 December 2023) were used to determine read quality. Illumina reads were utilized for PacBio sequence error correction using Pilon (v.1.23) [81], and read-based SNP discovery as described below. The resulting contigs were evaluated with QUAST (v.5.2.0 + Galaxy1) [82]. The chromosomal *oriC* (http://tubic.tju.edu.cn/Ori-Finder/, accessed on 10 December 2023) [83] and plasmid *repA* genes were designated as the zero point of the closed molecules, prior to annotation, using the NCBI Prokaryotic Genome Annotation Pipeline (PGAP) [84].

### 2.3. Pathogenome Make-Up and Virulence Complement

Chromosomes and plasmids of the strains, TT12A and TT12B, were comprehensively analyzed and visualized in Blast Ring Image Generator BRIG (v.0.95) [85]. Serotypes in the assembled genomes were confirmed in silico using the EcOH database [86] in ABRicate (Galaxy v.1.0.1); (https://github.com/tseemann/ABRicate, accessed on 10 December 2023) with the options –minid 80 –mincov 80 in Galaxy [80]. The average nucleotide identities (ANI) for the chromosomes and pO157 plasmids using the *E. coli* strain TT12A as designated reference were calculated with FastANI (Galaxy v.1.3), based on MinHash mapping [87]. Clade typing was performed according to [88]. Clades and subgroups were assigned by in silico interrogation of the allelic status of 89 core genome-single nucleotide polymorphisms (cgSNPs) in the assembled genomes using a custom workflow on Galaxy [80], which was informed by eight definitive polymorphic positions [89,90]. Lineages were assigned according to published protocols [91]. Chromosomal repeats were identified with FindRepeats (v.1.8.2 + Galaxy1) [92,93]. Virulence and antibiotic resistance genes were identified using VFDB [94] and ResFinder (https://cge.cbs.dtu.dk/services/ResFinder/, accessed on 10 December 2023) [95], respectively. Prophage regions including intact, partial, or remnant prophages were identified using PHASTER [96,97]. The mechanism of phage insertion can create direct repeats (DR), hence insertion sites were investigated for DR and attachments sites (*att*) using NUCmer (v.4.0.0rc1 + Galaxy2) [98] and BLASTn [99]. Prophages of interest were manually curated with BLASTn/p against the non-redundant NCBI databases [99] and visualized in Easyfig (v.2.2.2) [100]. Insertion sequence (IS) elements were identified and curated using ISEScan (v.1.7.2.3 + Galaxy0) [101]. Genomic islands (GI) were detected with IslandViewer4 [102,103,104]. Plasmid incompatibility groups were identified and analyzed with MOB-Typer (v.3.0.3 + Galaxy0) [105].

### 2.4. Core Genome SNP Phylogeny

To place strains TT12A and TT12B into their phylogenomic context, a custom-built cgSNP discovery pipeline [68,106,107], implemented on Galaxy [80], was applied. The chromosomal core genome is defined as a set of genic and intragenic regions that are not repeated and do not contain mobile elements, such as phages, genomic islands, IS elements, or plasmids, which evolve at different rates and are not indicative of evolutionary relationships. These regions were determined in the designated reference chromosome of *E. coli* strain EC4115 (GenBank accession: CP001164) [106] as described above. All mobile genetic elements were excluded from SNP discovery. Illumina reads were used for read-based SNP discovery. The modular pipeline contained the following workflow steps: (i) SNP discovery and typing Illumina reads were used for read-based SNP discovery and aligned to the designated reference with BWA–MEM (Galaxy v.0.7.17.2) [108]. The resulting alignments were processed with FreeBayes (Galaxy v.0.4.1.0) [109] with the following threshold settings: mapping quality 30, base quality 30, coverage 10, and allelic frequency 0.75. For contig-based discovery, assemblies were aligned to the O157:H7 strain EC4115 reference molecules using NUCmer [98], followed by SNP prediction with delta-filter (v.4.0.0rc1 + Galaxy2) and show-snps distributed with the MUMmer package (v.4.0.0rc1 + Galaxy2) [98,110]. The resulting SNP panel for each of the query genomes was used for further processing; (ii) SNP validation and filtering Catalogued SNPs from each genome were merged into a single SNP panel and SNPs located within the identified excluded regions were removed, as well as low-quality alignments or misalignments, non-uniformly distributed regions, and variant insertions and deletions (InDels), as previously described [106,107,111]. SNPs were further curated by extracting the surrounding 40 nucleotides (nt) for each predicted SNP in the reference genome, followed by BLASTn of these fragments against the query genomes. SNPs with missing information (“no hits”) or multiple hits were filtered out as well as ambiguous nucleotides; (iii) SNP annotation and chromosomal distribution The functional effects of SNPs were inferred from the reference genome annotation. Identified SNPs were classified into genic or intergenic by mapping the SNPs to the reference genome. The SNP-matrix tables were manipulated with Query Tabular Tool (Galaxy v.3.3.0] [112]; (iv) SNP phylogeny The curated panel of high-quality SNPs served as a basis for phylogenetic reconstruction by maximum parsimony with PAUP (v.4.0) [113] with 100,000 bootstrap replicates. The majority-rule consensus SNP tree was visualized in Geneious (v.2022.2) [114] and decorated in iTol (v.6.5.8) [115]. Calculation of the consistency index (CI) in Mesquite (v.3.6) [116] for each SNP allowed us to identify parsimony-informative SNPs and flag homoplastic SNPs as previously described [68,106,107,111,117,118]. Locally collinear blocks between TT12A and TT12B chromosomes and plasmids were identified with progressive Mauve (v.2.4.1) [119] with the default settings in Geneious (v.2022.2) [114]. Subtle sequence disambiguities comprising SNPs and InDels between the respective molecules were identified using “Find Variations/SNPs” in Geneious. A prediction of protein stability changes for single-site mutations was analyzed with MuPro (v.1.0) [120].

### 2.5. Core Genome MLST

The closed genomes of the representative O157:H7 strains (Supplemental Table S1) were imported into SeqSphere+ (v.8.3) (Ridom GmbH, Münster, Germany) for gene-by-gene alignment, allele calling, and comparison [121]. A core genome MLST (cgMLST) schema was developed using the closed chromosome of *E. coli* K-12 substrain, MG1655, (GenBank accession U00096) [122] as a seed and queried against 11 closed genomes representing the 9 distinct O157:H7 phylogenetic clades [68,88,106,107]. Core and accessory MLST targets were identified according to the inclusion/exclusion criteria of the SeqSphere+ Target Definer. The allele information from the defined core genome gene of the queried strains was used to establish phylogenetic hypotheses using the minimum-spanning method [123,124] with default settings in Ridom SeqSphere+ (v.8.3).

### 2.6. Bacterial Growth, Phage Induction, and Cell Viability

All experiments were executed with two biological replicates. TT12A and TT12B strains were cultured overnight (o/n) at 37 °C with shaking (220 rpm) in LB medium. Bacterial o/n cultures were diluted to an OD_600_ of 0.03 in fresh LB medium and grown at 37 °C with shaking (220 rpm) to early-log phase (OD_600_~0.3) and then divided into two subcultures, LB and LB + Mitomycin C (MMC). Triggering the RecA-dependent SOS response with MMC constitutes a major pathway of Stx phage induction and mobilization [125]. Subculture LB + MMC was supplemented with MMC (Sigma-Aldrich, Saint Louis, MO, USA) at a final concentration of 0.5 μg/mL to mobilize carried prophages, while subculture LB was used to evaluate spontaneous prophage mobilization. Growth curves were recorded in a 96-well plate (Corning 3370, Corning Inc., Corning, NY, USA) at OD_600_ on a BioTek Synergy H1 plate reader (BioTek Instruments, Inc., Winooski, VT, USA) for 16 hrs at 10 min intervals to assess prophage-induced bacterial lysis.

### 2.7. Prophage Profiling and Gene Expressions

PCR primer sequences, conditions, and amplicon lengths are provided in Supplemental Table S2. PCR was performed on gDNA preparations using the boiling extraction method [126] for strains TT12A and TT122B, processing three cultures each for the characteristic TT12A- and TT12B-specific morphology [62]. To determine the orientation of an inversion in the shared *Enterobacteria* phage SfI-PP2, primers were designed with the NCBI primer design tool [127]. PCR-amplicons were separated on a 1.5% agarose gel at 120V and examined in the GelDoc EZ Gel Imaging System and ImageLab (v.6.1) (BioRad, Hercules, CA, USA).

### 2.8. Mobilization of TT12A-Specific Prophages

To assess TT12A-specific phage mobilization upon MMC-induction, LB and LB + MMC subcultures were grown for 6 hrs at 37 °C with shaking (220 rpm) and then centrifuged at 5000× *g* for 10 min. Supernatants were filtered through low-protein-binding 0.22 μm pore size membrane filters (Millex-GP; Merck Millipore Ltd., Burlington, MA, USA) and treated with DNase I (Invitrogen, Waltham, MA, USA) for 15 min to remove bacterial gDNA. Phage DNA was extracted from the lysate using the QIAamp DNA Mini Kit (Qiagen Inc., Valencia, CA, USA), and eluted with 50 μL nuclease-free water. Phage mobilization was determined by qPCR targeting the phage-borne toxin genes *stx1* and *stx2* as well as ΦPP10-carried *nleL* gene on the StepOne Real-Time PCR System software (v 2.3) (Applied Biosystems, Foster City, CA, USA). Statistical significance was determined using Prism (v.9.5.0) (GraphPad Software, San Diego, CA, USA) with two-way ANOVA with Sidak’s multiple comparisons test to compare non-induced to MMC-induced conditions.

### 2.9. Gene Expressions

Transcripts were quantified relative to the endogenous *tufA* gene by RT-qPCR [128]. Cultures were grown in LB and LB + MMC for 6 hrs at 37 °C with shaking (220 rpm) then centrifuged (5000× *g*, 10 min). Cell pellets were used for total RNA purification using the PureLink RNA Mini kit (Invitrogen, Waltham, MA, USA). RNA quantity and quality were measured with the NanoDrop ND-1000 Spectrophotometer (Thermo Fisher Scientific, Waltham, MA, USA). Total RNA was treated with amplification grade DNase I (Invitrogen, Waltham, MA, USA), and reverse transcribed using the RevertAid H Minus First Strand cDNA Synthesis Kit (Thermo Fisher Scientific, Waltham, MA, USA). Targets for qPCR were the toxin genes *stx1* and *stx2* and the SOS-regulator *recA*. PCR reactions were performed on the StepOne Real-Time PCR System (Applied Biosystems, Foster City, CA, USA) using GoTaq qPCR Master Mix (Promega, Madison, WI, USA). Primer sequences, RT-qPCR conditions, and amplicon lengths are provided in Supplemental Table S2. Statistical significance was determined using Prism (v.9.5.0) (GraphPad Software, San Diego, CA, USA) with two-way ANOVA with Sidak’s multiple comparisons test to compare results of non-induced to MMC-induced conditions for each strain.

## 3. Results and Discussion

### 3.1. Pathogenome Architectures and Mobilome Inventories of TT12A and TT12B

**Figure 1:**
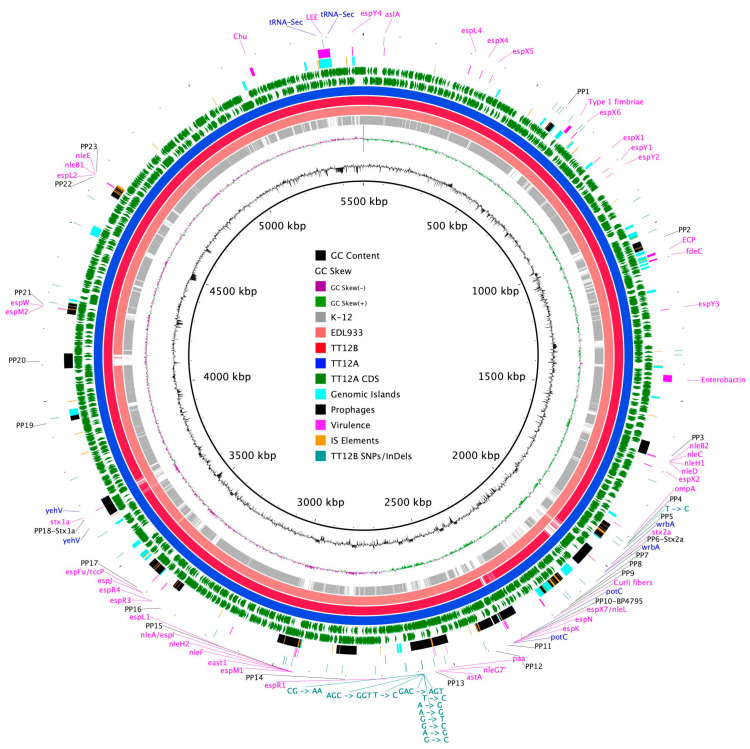
Chromosome comparison of TT12A and TT12B BRIG comparison of the Stx(+) TT12A and Stx(−) TT12B genome architecture and gene content referenced to the larger Stx(+) genome. The comparison is further extended to include the EDL933 strain, clade 3.12, and the K-12 *E. coli* strain. CDSs are presented as arrows on the +/− strands, and functional annotations for the shared and three TT12A-specific prophages, virulence, and resistance genes, along with polymorphisms differentiating the two strains, are highlighted as shown in the legend. Chromosomal synteny in K-12 is disrupted by multiple prophages and other MGEs.

**Figure 2:**
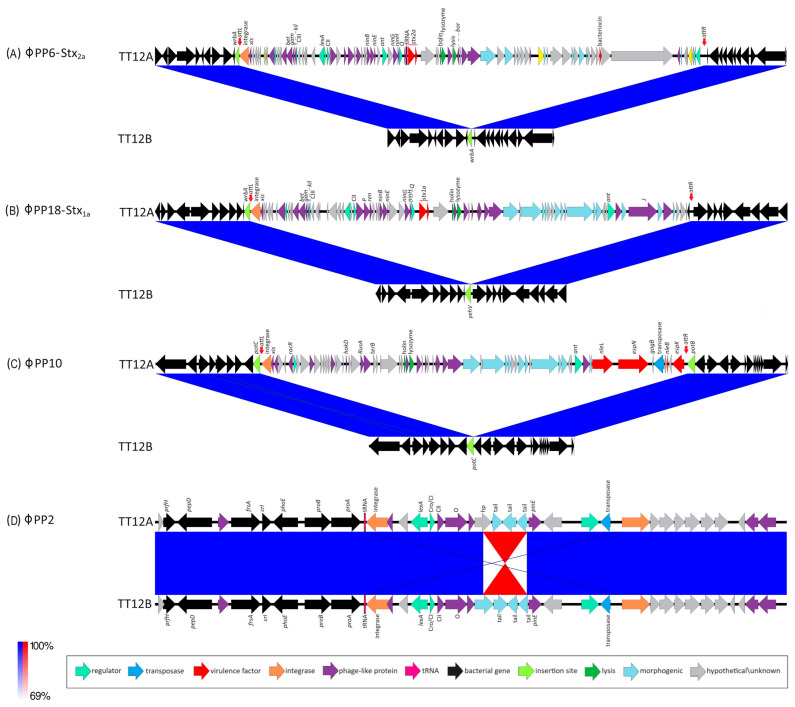
Prophages and Stx-status of TT12A and TT12B BLASTn-based comparison of the prophage inventory and polymorphisms visualized in Easyfig. Strains are differentiated by the presence of three additional ΦStx- and Φnon-Stx prophages in the larger (*stx*+) TT12A strain at the following loci: (**A**) ΦStx_2a_-phage at *wrbA*: This locus is unoccupied in TT12B, while in strain A, a 63,250 kb ΦStx_2a_-prophage is inserted at *wrbA*, a preferred target locus for ΦStx_2a_-phage insertion in O157:H7. (**B**) ΦStx_1a_-phage at *yehV*: This locus is unoccupied in TT12B, while a 53,637 kb ΦStx_1_-prophage is inserted at *yehV*, a preferred target locus for ΦStx_1a_-phage insertion in O157:H7. (**C**) ΦPP10-*Enterobacteria* phage BP-4795 at *potC*: This locus is unoccupied in TT12B, while a 49,073 kb prophage is inserted at *potC*, a known target for phage insertion in O157:H7. (**D**) ΦPP2-*Enterobacteria* SfI phage: Chromosome assemblies feature an inversion within the shared ΦPP2-*Enterobacteria* SfI prophage.

**Figure 3:**
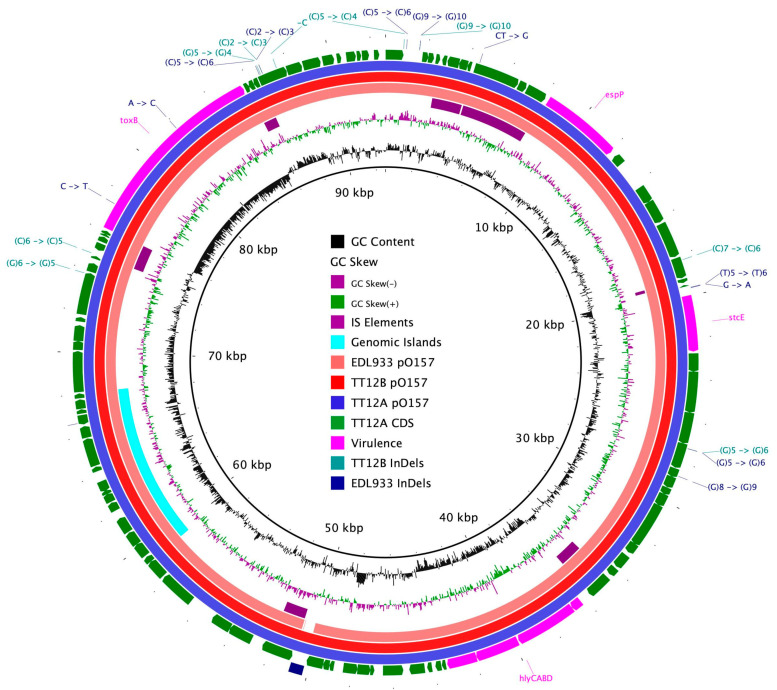
Comparison of clade 3 pO157 plasmids BRIG comparison of the pO157 plasmid architecture and gene content. CDSs are presented as arrows on the +/− strands, and functional annotations for notable virulence determinants, such as EHEC hemolysin (*hlyCABD*), the serine protease (*espP*) along with plasmid differentiating polymorphisms, including SNPs and InDels, are highlighted as shown in the legend.

The proposed isogenic status for the O157:H7 isolates TT12A (*stx+*) and TT12B (*stx−*), was originally inferred from the PCR-based interrogation of the *stx* locus, along with a similar PFGE fragmentation pattern [62]. To reassess the isolates’ relationship, their closed genomes were subjected to high-resolution whole genome sequence typing (WGST) [107,129]. Due to the homogeneity of prophage content and other repeats, *E. coli* O157:H7 genomes are known to assemble into fragmented drafts when only short-read sequencing technologies are applied [130,131]. In response, we used PacBioRS long-read sequencing followed by error correction with Illumina short-reads. The resulting high-quality genomes allowed us to catalogue strain-differentiating sequence and structural polymorphisms at a high degree of phylogenetic resolution. Strain-associated metadata and genome statistics for the chromosomes and carried lineage-specific pO157 virulence plasmids are provided in Supplemental Table S1. Major drivers of the pathogenome diversification in the O157:H7 lineage are mobile genome elements and more prominently, the individual prophage contents [106,107,132,133,134,135,136]. As evident in the chromosome comparison in Figure 1, the TT12A and TT12B backbones are largely conserved and syntenic with an average nucleotide identity (ANI) of 99.99%. In comparison to non-pathogenic *E. coli* K12-type strains, O157:H7 acquired ΦStx and non-Stx prophages resulting in widespread genetic mosaicism [132,137,138,139]. Bacteriophages target conserved chromosomal loci and undergo evolutionary acquisition, loss, and dissemination, which collectively shape a strain’s individual gene inventory and pathogenicity traits [140,141,142]. A substantial proportion of the TT12A and TT12B chromosomes is made up of prophages, accounting for 16% and 13.1%, respectively, of the total chromosomal sequence information. 21 phages are shared; however, strain TT12A (5,501,009 bp) is distinguished from strain TT12B (5,335,866 bp) by the presence of three additional phages, ΦStx_1a_, ΦStx_2a_, and non-Stx *Enterobacteria* phage ΦBP-4795 prophage. The latter is partly homologous to the 57,930 bp ΦStx_1_-prophage carried by the *E. coli* O84:H4 strain with an average nucleotide identity of 45.3% [143]. The direct evolutionary relationship of these Stx/non-Stx phages is unknown; however, alterations of the *stx* locus, resulting in confined loss or deletion of the entire phage, has been described in diverse STEC lineages [68]. The absence of these three prophages, ΦStx_1a_, ΦStx_2a_, and non-Stx *Enterobacter* phage ΦBP-4795, in the strain TT12B results in a 165,143 bp larger genome of strain TT12A (Figure 1, Supplemental Table S3). The three strain-differentiating prophages along with an inversion in the shared ΦPP2 are visualized in their chromosomal context in Figure 2. In analogy to the chromosomes, the carried pO157 plasmids have an ANI of 99.98%, and differ in size by only 3 bp, again indicative of a close phylogenetic relationship of these strains (Figure 3).

### 3.2. Phylogenomic Position of TT12A and TT12B within the O157:H7 Lineage

**Figure 4:**
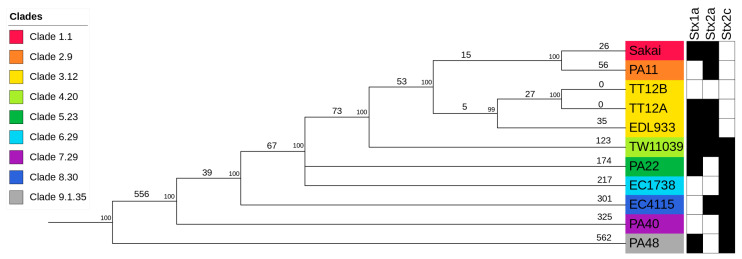
Phylogenomic position of TT12A and TT12B within the O157:H7 serotype Genome comparisons of TT12A and TT12B and the representative strains from clades 1 to 9 O157:H7 genomes yielded a total of 2,654 SNPs when referenced to strain EC4115. The tree shown is the majority-consensus tree of three equally parsimonious trees with a CI of 0.998. Trees were recovered using a heuristic search in PAUP with 100,000 bootstrap replicates. The tree is rooted to the clade 9 strain, PA48, visualized and decorated in iTol. Nodes are color-coded according to clade, and numbers of separating SNPs are shown. The prevalence of Stx-subtypes is indicated by black (*stx*+) and white (*stx−*) boxes.

In silico genotyping classified TT12A Stx(+) and its co-isolated TT12B Stx(−) strains as Sequence Type ST11, Lineage I, Clade 3.12 isolates (Supplemental Table S1) [88,91,121,144]. To place the strains into the broader context of O157:H7/NM evolution [51,52,53,54,55,56], we constructed a phylogenetic hypothesis based on reference-based cgSNP discovery including representative O157:H7 strains for the nine distinct clades [68,88,106,107] (Supplemental Table S4). As evident in Figure 4, the overall tree topology reflects the general understanding of O157:H7/NM evolution from an enteropathogenic *E. coli* (EPEC) O55:H7 progenitor [51,52,53,54,55,56,145]. Clade 3.12 strains TT12A, TT12B, and EDL933 form a cluster, and strains TT12A and TT12B were found to be indistinguishable on the chromosomal cgSNP level [62]. This intimate relationship is further mirrored in the cgMLST analysis, with no allelic changes observed (Supplemental Figure S1, Supplemental Table S4), and along with the SNP information being consistent with an isogenic status. The strains’ intimate relationship is also reflected in the IS element profiles. In total, ISEScan detected 87 IS elements and categorized them into 14 families and 27 clusters (Supplemental Table S3). The strains feature identical chromosomal or plasmid-borne profiles, not considering the two phage-borne IS elements that are part of the TT12A-specific *Enterobacteria* phage ΦBP-4795-PP10 (Figure 1, Supplemental Table S3), supporting an intimate relationship between these two strains. To build the clade-inclusive cgSNP phylogeny (Figure 4), we excluded SNPs within mobile elements and repeats, as the alignment of homologous regions of different evolutionary origins can introduce false positive signals and ultimately, phylogenetic inaccuracies [106,107]. We thus used an alternative approach for these closely related isolates and processed all collinear blocks in their genomes to record the SNPs and InDels. This resulted in the detection of 16 chromosomal intragenic SNPs, all situated within two prophages, while no SNPs were recorded on the pO157 (Supplemental Table S4). In this context, we note that plasticity in the ΦStx-phage complement of O157:H7 and other STEC serotypes is well established [138]. Particular variations within the ΦStx_2a_-phages have been associated with altered toxin production capabilities [146,147,148,149]. Exploring the effects of such phage-to-phage variants on phage–host interactions or pathogenesis could deepen our understanding of the evolution of O157: H7 virulence. A single nonsynonymous (ns) SNP was detected in the shared ΦPP4-phage, while 15 SNPs were located within a single gene on the carried TT12A-ΦPP13 and TT12B-ΦPP11 prophages that code for the host specificity protein J [150]. Though we cannot delineate the physiological effects of these variants, we note that 80% of SNPs are non-synonymous and are predicted to decrease protein stability in TT12B [120] with potential impacts on phage biology.

In addition, we detected 65 strain-differentiating InDels, 56 of which are chromosomal (Supplemental Table S4, Figure 1 and Figure 3). The majority, 38 chromosomal and 8 plasmid-borne InDels, are located within homopolymer repeats, which are prone to dynamic expansion or shrinkage during short-term evolutionary terms [151]. We also note here that 37 of the chromosomal and 1 of the plasmid InDels are associated with mobile genetic elements, including prophages, IS elements, and genomic islands (Supplemental Table S4).

### 3.3. Comparison of Virulence and Resistance Determinants

**Figure 5:**
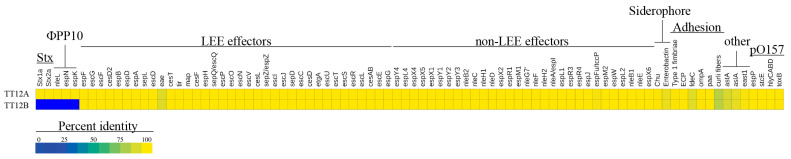
Virulence determinants in TT12A and TT12B A heatmap visualizing the percentage identities for each virulence determinant identified in TT12A and TT12B. Apart from Stx-phage contributed toxins and *nleL*, *espN*, and *espK* genes on ΦPP10 *Enterobacteria* phage BP-4795 only present in TT12A, the strains’ virulence profiles are similar.

We surveyed the TT12A and TT12B genomes for chromosomal and plasmid-borne virulence and resistance loci. Neither strain carries antibiotic resistance genes, other than the chromosomal broad-spectrum multidrug resistance efflux pump, MdfA [152], commonly found in *E. coli* [153,154,155]. Not taking into account the TT12A-specific phage inventory, comprising the ΦStx_1_/_2a_-phages and several T3SS effectors *nleL*, *espN*, and *espK* on the *Enterobacteria* phage ΦBP-4795-PP10 (Figure 1), the strains’ virulence profiles are indistinguishable, showing a characteristic O157:H7 virulence repertoire [156,157] (Figure 5, Supplemental Table S3). The virulence factors include the locus of enterocyte effacement (LEE) at the tRNA-Sec (Figure 1) [23,158,159,160,161] which causes attaching and effacing (A/E) lesions [22,23]. This pathogenicity island encodes T3SS components, such as regulators, chaperones, and LEE/non-LEE effectors [22,24,25]. Among the LEE-encoded proteins is intimin (*eae*-*γ1*) [162,163], an outer membrane adhesin that facilitates intimate bacterial attachment to the host’s intestinal cells [24,158,164,165,166,167] (Figure 5). Variation in the architecture and coding capabilities of the lineage-specific virulence pO157 plasmid has provided insight into O157:H7 evolution [68,168,169,170,171]. The TT12A (92,710 bp) and TT12B (92,707 bp) plasmids are highly conserved with a size difference of only 3 bp, supporting an intimate relationship between these two strains [18,68,168,172,173] (Figure 3 and Figure 5). Loss of pO157 has been linked to diminished virulence in O157:H7 strains [174]. Characteristic plasmid-borne virulence determinants are a type II secretion system metalloprotease (*stcE*), hemolysin (*hlyCABD*), enterohemolysin (*ehxA*), type V-secreted serine protease (*espP*), and adhesin (*toxB*) [18,172,173,175,176,177] (Supplemental Table S3).

### 3.4. Prophage Content and Genomic Basis of the Attenuated Stx(−) Status

Toxin production and organ toxicity is dependent on the carried *stx* suballeles [48,178,179,180]. Diverse *stx*-phage combinations are found in the O157:H7 lineage [68,107,133,147]. A genetically altered nonstandard *stx*-locus, such as through IS element disruption [68], might not amplify with generic PCR primers and thus can create false-negatives [42,62,143,181]. The availability of closed genomes is thus critical to describe the genomic basis of the absence of *stx* genes in TT12B. Strain TT12A carries two Stx-prophages, ΦStx_2a_-PP6 (63,250 bp) (Figure 2A) and ΦStx_1_-PP18 (53,637 bp) (Figure 2B) inserted into *wrbA* and *yehV* [182,183] (Figure 1), respectively, which are the preferred insertion targets of these particular Stx-phage subtypes [68,106,138]. In mice, Stx_2a_ confers up to 400x times higher toxicity than Stx_1_ [11,28,45,46,47,48,49]. Both Stx B-subunits bind to the Globotriaosylceramide receptor, Gb3, on the eukaryotic cell. The Stx_2a_–Gb3 binding, however, is weaker in comparison to Stx_1a_–Gb3 [147,184]. Consequently, the Stx_1_/_2a_ toxin status of TT12A may indicate lower toxicity in comparison to *stx_2a_*-only strains [147,180,184,185,186]. Furthermore, we quantified the expression levels of the co-carried toxin variants. In cultures of TT12A treated with MMC, both phages were responsive, and we recorded a 4.4-fold and a 3.4-fold increase in *stx_2a_* and *stx_1a_* transcripts, respectively (Supplemental Figure S2). The ΦStx_2a_ phage is of the Eru-2 type, characterized by the absence of the lambdoid O and P genes, while the ΦStx_1a_ identifies as the lambdoid–Eru type [148,149] (Figure 2). The ΦStx_1a_ phage features a γ-replicase, when applying a replicase P-informed subtyping schema [146]. However, the ΦStx_2a_ phage is not typable, showing only 53% identity to β-replicase subtype. TT12A is further distinguished by the presence of *Enterobacteria* phage ΦBP-4795-PP10 (49,073 bp) at *potC* (Figure 2C)*,* a known phage insertion site in O157:H7 and other STEC serotypes [187,188,189]. In silico comparison further identified an 1833 bp inversion within the shared incomplete ΦPP2-prophage (Figure 2D, Supplemental Table S3). Further PCR interrogation, however, revealed that this polymorphism is not strain-specific; and both orientations were detected in cultures of TT12A and B (Supplemental Figure S3). This inversion affects a single gene, annotated as a phage tail protein in the TT12B assembly (locus tag: E5F07-04800) and a hypothetical protein in TT12A (E5F08-04795) (Figure 2D).

### 3.5. Differences in Growth Phenotypes and Impact of the Prophage Inventories

We recorded growth in LB and under phage-mobilizing conditions in LB + MMC to determine the degree of prophage-induced lysis in TT12A and TT12B. Increased expression of *recA* post MMC treatment confirmed the successful SOS response activation in both cultures (Supplemental Figure S4A) [125,190,191,192]. Culture growth of TT12A is considerably affected through phage lysis, unlike strain TT12B (Supplemental Figure S4B). The different growth phenotypes in the MMC-treated cultures thus seem to be mediated by the three TT12A-specific ΦStx_1a_/_2a_/non-Stx phages, all of which are known or predicted to be lytic in the case of *Enterobacteria* phage ΦBP-4795-PP10 (Figure 2, Supplemental Table S3). Even though another seven lytic phages are predicted as part of the shared phage complement (Supplemental Table S3), these phages are likely not mobilized through the SOS response pathway activation, as evident in the similar growth of TT12B in LB and LB + MMC media (Supplemental Figure S4B).

## 4. Conclusions

Whole genome sequence typing has proven to be invaluable for the identification and strain attribution of near clonal *E. coli* pathogen populations [40,107,193,194]. Availability of high-resolution closed genomes allowed us to record subtle strain-level sequence and structural polymorphisms with high phylogenetic accuracy, demonstrating an isogenic relationship of these Stx(+/−) TT12A and TT12B isolates. The number of strain-differentiating SNPs is similar to the range reported for clonal O157:H7 outbreak strains [40,106,107,111,195]. However, SNP data on its own are clearly insufficient to infer clonality in microbes without further assessing changes in genome structure and content. Dynamic phage acquisition and loss resulted in the strain-specific ΦStx and non-ΦStx prophage content, which may have occurred in a single or separate evolutionary events likely triggered by the mobilization and ultimately, loss of these phages. STECs have been intentionally cured from their Stx-phages by antibiotic or MMC addition to the growth medium [63,68,196]. In this context, it is noteworthy that excised copies of the three TT12A-differentiating prophages ΦStx/non-Stx phages, absent in TT12B, were significantly increased when grown in the phage-inducing LB + MMC media (Supplemental Figure S5). Additionally, none of the insertion sites occupied in TT12A showed scarring that would indicate a former phage presence in TT12B. Alternative evolutionary scenarios can explain the Stx-phage absence in atypical O157:H7 strains, such as TT12B [51,68,197]. We can only speculate about the events that gave rise to the TT12A and TT12B variants. Given the fact that these strains originate from a patient suffering from hemorrhagic colitis [62] along with the strains’ established intimate phylogenetic relationship, the secondary loss of both ΦStx_1_/_2_ prophages along with *Enterobacteria* phage ΦBP-4795-PP10 in TT12B during the course of infection, in a singular or in multiple events, seems likely. The ratio of Stx(+) to non-shigatoxigenic isolates is not known; however, dynamic loss of Stx-phages and potential re-acquisition may cause transitional *stx*(+/−) shifts in pathogenic potential [68,69,74]. Bacteriophages control diverse bacterial biological functions. Stx has a dual role in human disease and spontaneous low-level Stx production is considered a form of bacterial altruism, promoting the toxin-dependent killing of eukaryotic predators and macrophages [29,198,199,200,201,202,203,204,205]. The ΦStx phage carriage has been associated with a number of virulence and fitness traits beyond Stx-production. The characterization of laboratory-engineered Stx-lysogens, often recovered in *E. coli* K12 backgrounds, indicated an impact on acid resistance, type III secretion, motility, and metabolism [206,207,208,209,210,211,212,213,214,215]. The analyzed Stx(+/−) isogen cultures and genomes provide an excellent model to further investigate the impact of ΦStx-phage carriage in a native O157:H7 genome background that is not accounted for in the K12-engineered Stx-lysogens [206,208,210,215]. Altogether, the genomic and phenotypic comparisons show that this isogen pair is intimately related, yet perhaps on a divergent evolutionary path. The role of the catalogued sequence and architectural polymorphisms, however, cannot be simply inferred from static genome comparison. Further investigations using transcriptomic and phenotypic profiling may provide greater insight into the role these variants may play in the physiology and pathogenicity of strains TT12A and TT12B.

## Data Availability

The sequence data sets generated and analyzed in this study have been deposited in the Sequence Read Archive (SRA) and GenBank at NCBI under BioProject PRJNA530317. Accessions for the reads, assembled annotated chromosomes, and pO157 plasmids of strains TT12A (CP038496, CP038497) and TT12B (CP038494, CP038495) [68] are provided in Supplemental Table S1. This table also includes strain-associated metadata and representative O157:H7 strains retrieved from NCBI in support of comprehensive analyses.

## Supplementary Materials

The following supporting information can be downloaded at: https://www.mdpi.com/article/10.3390/microorganisms12040699/s1, Figure S1: Core genome MLST, Figure S2: Expression of *stx* genes, Figure S3: PCR interrogation of inversion boundaries and directionality in ΦPP2, Figure S4: Growth phenotypes, Figure S5: Mobilization pattern of TT12A-specific phages; Table S1: Strain-associated metadata and sequence accessions, Table S2: Primers used in this study, Table S3: Genome features and contents, Table S4: Core genome SNP and MLST loci.
