# Supplementary material for: Pathogenomes of Shiga Toxin Positive and Negative Escherichia coli O157:H7 Strains TT12A and TT12B: Comprehensive Phylogenomic Analysis Using Closed Genomes
Source: Microorganisms. 2024 Mar 29;12(4):699. doi: 10.3390/microorganisms12040699 (PMC11052207; doi:10.3390/microorganisms12040699)
Supplement: Supplementary file 1 [file microorganisms-12-00699-s001.zip › Supplemental_Figures.pdf]

Supplemental Figures

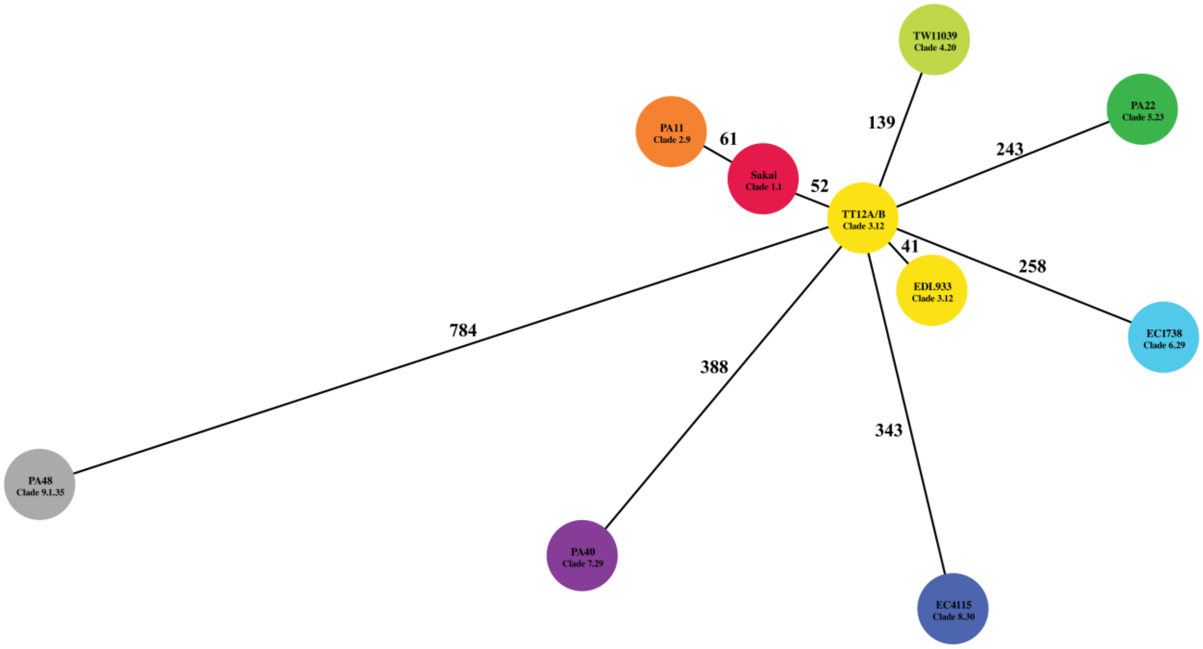

Figure S1. Core genome MLST. Allelic changes were detected in the genomes of TT12A and TT12B and representative strains from clades 1 to 9 O157:H7 using *E. coli* strain K12 subst. MG1655 as seed in SeqSphere+. The shared gene inventory was determined at 4,056 genes comprising 3,224 core and 832 accessory loci according to the inclusion/exclusion criteria of the SeqSphere+ Target Definer. Numbers on connecting branches indicate the number of genes with differing allele status.

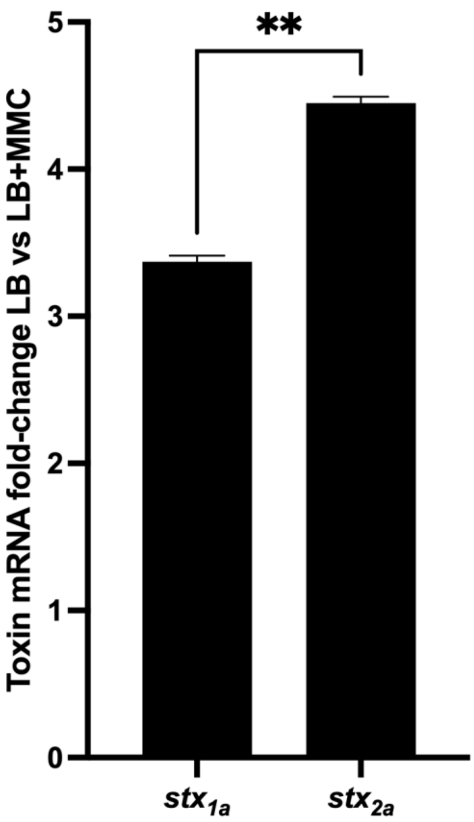

Figure S2. Expression of *stx* genes. RT-qPCR based relative gene expression upon MMC-induction is quantified in fold-change for the toxin genes, stx_{1a} and stx_{2a} in strainTT12A. Culture TT12A shows significantly elevated expressions of both the stx_{1a} and stx_{2a} genes in phage-mobilizing LB+MMC condition when compared to growth in LB, also statistically significant more stx_{2a} transcripts were made relative to stx_{1a}. Statistical significance for two biological replicates is reported as *P < 0.05; **P < 0.005; ***P < 0.0005; ****P < 0.00005.

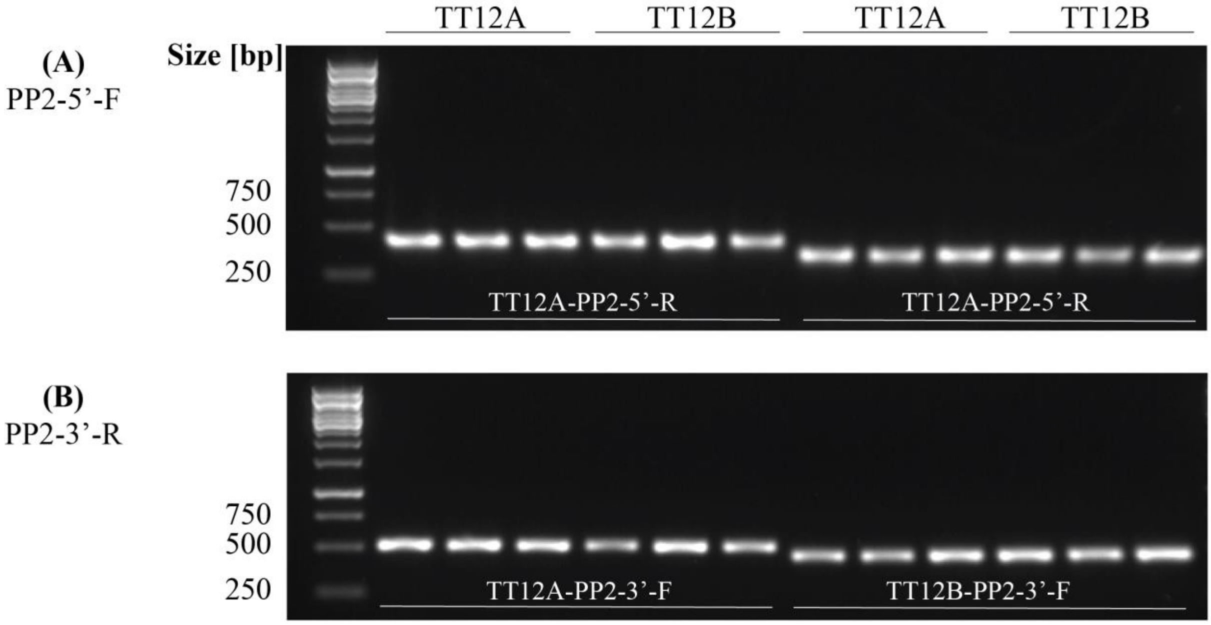

Figure S3. PCR-interrogation of inversion boundaries and directionality in ΦPP2 Two distinct PCRs were performed using two anchor primers (**A**): PP2-5'-F and (**B**): PP2-3'-R located in the inversion adjacent core region. For the TT12A assembly, primers were designed to target a hypothetical gene (E5F08-RS27785) and a phage tail gene (E5F08-RS04765). For the TT12B assembly, primers target two phage tail genes (E5F07-RS04740, E5F07-RS04755).

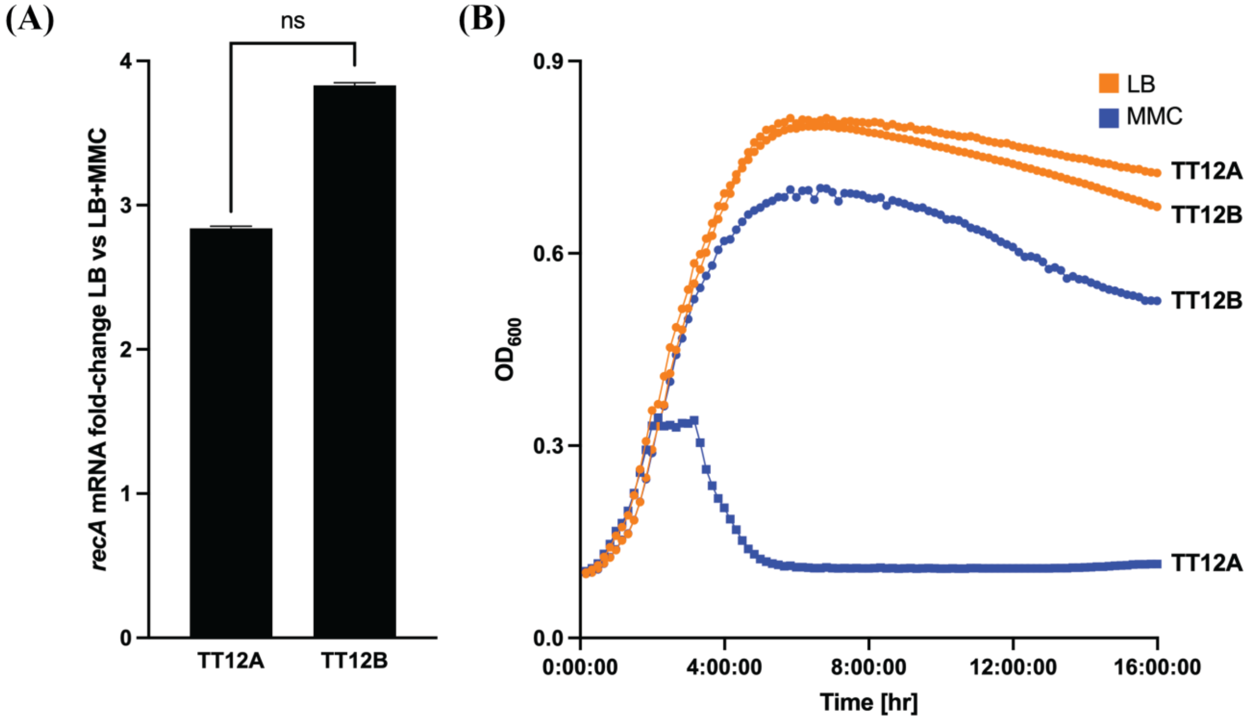

Figure S4. Growth phenotypes. Growth of TT12A and TT12B cultures in LB and under phage-mobilizing conditions in LB+MMC was recorded for 16 hrs. (**A**) RT-qPCR based relative gene quantification of the SOS key regulator *recA* six hrs post-MMC treatment shows SOS-response activation in both TT12A and B cultures. Statistical significance for two biological replicates is reported as $*P < 0.05$; $**P < 0.005$; $***P < 0.0005$; $****P < 0.00005$. (**B**) Cultures grown in LB show similar growth patterns. Upon MMC-induction (time=0 hr), TT12A cultures show impaired growth due to phage lysis when compared to TT12B.

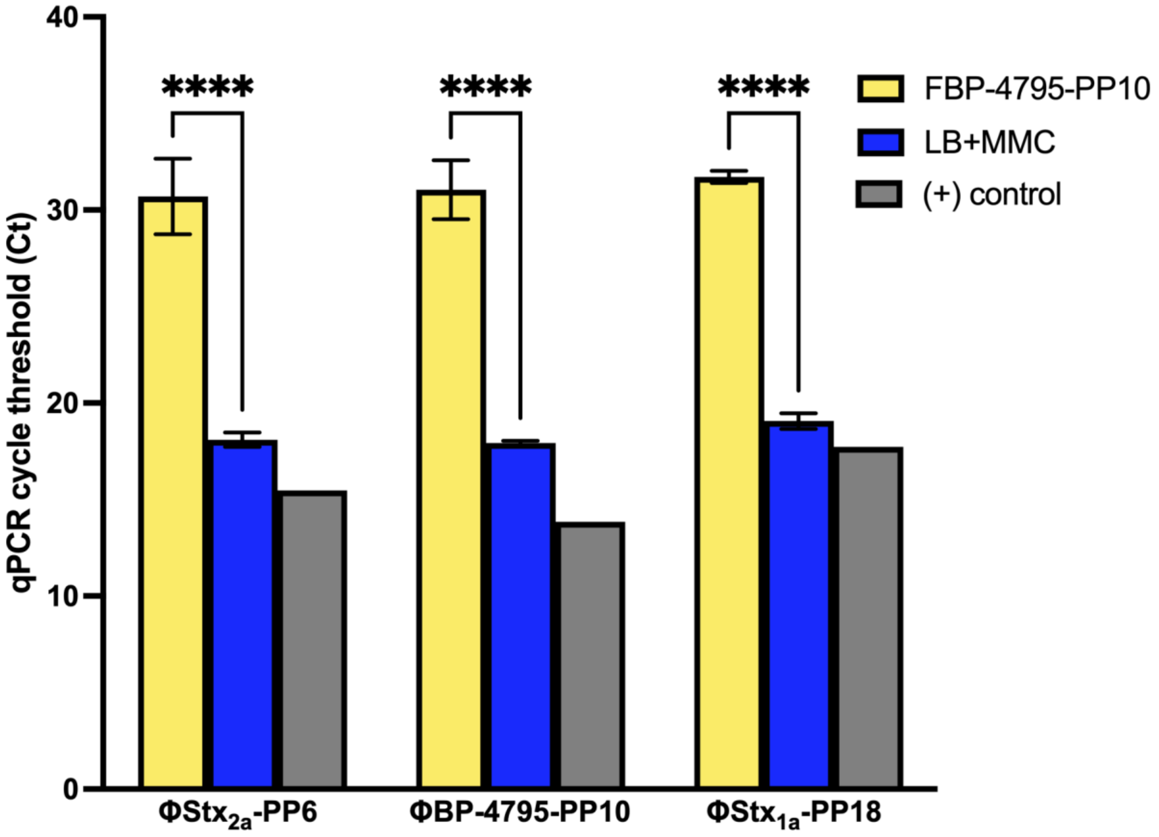

Figure S5. Mobilization pattern of TT12A-specific phages The copy numbers of TT12A-specific excised Stx_{1a}-, Stx_{2a}-, and PP10-prophages were calculated by qPCR in LB and LB+MMC conditions. The abundance of phage DNA and bacterial gDNA, used as a positive control, are displayed as Ct values. Statistical significance for two biological replicates is reported as ***$P < 0.0005$.
